# Bacterial Hsp90 Facilitates the Degradation of Aggregation-Prone Hsp70–Hsp40 Substrates

**DOI:** 10.3389/fmolb.2021.653073

**Published:** 2021-04-15

**Authors:** Bruno Fauvet, Andrija Finka, Marie-Pierre Castanié-Cornet, Anne-Marie Cirinesi, Pierre Genevaux, Manfredo Quadroni, Pierre Goloubinoff

**Affiliations:** ^1^Department of Plant Molecular Biology (DBMV), University of Lausanne, Lausanne, Switzerland; ^2^Department of Ecology, Agronomy and Aquaculture, University of Zadar, Zadar, Croatia; ^3^Laboratoire de Microbiologie et de Génétique Moléculaires, Center de Biologie Intégrative, CNRS, Université de Toulouse, Toulouse, France; ^4^Protein Analysis Facility, University of Lausanne, Lausanne, Switzerland

**Keywords:** chaperones, DnaK, DnaJ, proteostasis, HslV, HtpG

## Abstract

In eukaryotes, the 90-kDa heat shock proteins (Hsp90s) are profusely studied chaperones that, together with 70-kDa heat shock proteins (Hsp70s), control protein homeostasis. In bacteria, however, the function of Hsp90 (HtpG) and its collaboration with Hsp70 (DnaK) remains poorly characterized. To uncover physiological processes that depend on HtpG and DnaK, we performed comparative quantitative proteomic analyses of insoluble and total protein fractions from unstressed wild-type (WT) *Escherichia coli* and from knockout mutants Δ*dnaKdnaJ* (ΔKJ), Δ*htpG* (ΔG), and Δ*dnaKdnaJ*Δ*htpG* (ΔKJG). Whereas the ΔG mutant showed no detectable proteomic differences with wild-type, ΔKJ expressed more chaperones, proteases and ribosomes and expressed dramatically less metabolic and respiratory enzymes. Unexpectedly, we found that the triple mutant ΔKJG showed higher levels of metabolic and respiratory enzymes than ΔKJ, suggesting that bacterial Hsp90 mediates the degradation of aggregation-prone Hsp70–Hsp40 substrates. Further *in vivo* experiments suggest that such Hsp90-mediated degradation possibly occurs through the HslUV protease.

## Introduction

Various stresses and mutations increase the propensity of labile cellular proteins to transiently unfold and convert into stably misfolded and aggregated conformers that have lost their dedicated biological function. Moreover, protein aggregates, especially early soluble forms, may be toxic to eukaryotic cells by causing membrane damage, the release of reactive oxygen species, leading to cell death, aging, and degenerative diseases (Lashuel and Lansbury, 2006; Hinault et al., 2011). Various chaperone orthologs from the conserved 70-kDa heat shock protein (Hsp70) and 90-kDa heat shock protein (Hsp90) families are present at concentrations on the order of tens of micromolar in all ATP-containing compartments of the eukaryotic cells (Finka et al., 2015). Typically assisted in the cytosol by the Hsp70–Hsp90 organizing protein (HOP), the Hsp70s and Hsp90s stand together at the center of a network of molecular chaperones, co-chaperones, and proteases controlling all aspects of cellular protein homeostasis. They assist *de novo* folding of nascent polypeptides, drive protein translocation, control polypeptide maturation, and assembly into functional complexes and target labile native and stress-damaged proteins to degradation by the proteasome or in bacteria, by similar ATP-dependent chaperone-gated proteases, such as FtsH, Lon, HslUV, and ClpXP (Finka et al., 2016; Hartl, 2017). Attesting for their centrality under both physiological and stress conditions, members of the Hsp90 and Hsp70 families are among the most abundant proteins of the human proteome, accounting for 2–5% of the total protein mass of some cells (Finka et al., 2015).

As suggested by their elevated basal levels in unstressed cells, Hsp90s and Hsp70s control a plethora of physiological processes, such as the conversion of active protein oligomers into transiently inactive subcomplexes, as in the case of clathrin cages, Inhibitor kappa B (IκB), and heat shock transcription factor 1 (HSF1) oligomers (Pratt et al., 2010; Finka et al., 2016). The downregulation or knockout mutations of Hsp90 genes dramatically affects cellular growth and reduces survival following various stresses, such as heat shock (Franzosa et al., 2011) and Hsp90 inhibitors induce cell death, rendering them possibly attractive targets for cancer therapy (Ayrault et al., 2009).

*Escherichia coli* cells express the multifunctional Hsp70, DnaK, and a single Hsp90, HtpG. Both sequence- and structure-wise, HtpG and DnaK share a very high degree of homology with their respective eukaryotic Hsp90 and Hsp70 counterparts, strongly suggesting that they carry similar collaborative physiological- and stress-related proteostasis functions in bacteria, mitochondria, and chloroplasts. The deletion of the single *dnaK* gene from *E. coli* causes a severe growth phenotype at 37°C and above, accompanied by the strong accumulation of insoluble protein aggregates (Mogk et al., 1999). Stable isotope labeling by amino acids in cell culture (SILAC)-based comparative proteomic studies showed that at 30°C, a significant number of aggregation-prone Hsp70 substrates are degraded in Δ*dnaKdnaJ* cells when compared to wild type (WT) (Calloni et al., 2012). In contrast, the deletion of the *htpG* gene has virtually no phenotype at 37°C and below, except for a deficiency in the clustered regularly interspaced short palindromic repeats (CRISPR)–Cas adaptive immunity (Yosef et al., 2011). Only at very elevated temperatures (42–46°C), *htpG* mutants exhibited mild growth retardation (Bardwell and Craig, 1988; Thomas and Baneyx, 1998) and a minor accumulation of aggregated proteins (Thomas and Baneyx, 2000). In cyanobacteria, the deletion of *htpG* only results in a mild phycobilisome assembly phenotype under physiological conditions (Sato et al., 2010) but has otherwise little measurable impact on their physiology. In *Shewanella oneidensis*, HtpG is required for growth under heat stress, as it stabilizes the essential protein TilS under these conditions (Honore et al., 2017). Yet, *htpG* remains highly conserved and is clearly expressed in most eubacteria thus far investigated, suggesting that a specific essential biological function of HtpG common to all prokaryotes, especially under stress, remains to be identified.

Quantitative proteomic studies offer absolute quantification of most of proteome of a cell and offer insights into chaperone mechanisms in proteostasis, in bacteria (Calloni et al., 2012), plant (Guihur et al., 2020), and mammalian cells (Geiger et al., 2012; Gat-Yablonski et al., 2016). Here, we addressed by label-free quantitative proteomic analysis of total and insoluble protein fractions from WT, Δ*dnaKJ* (ΔKJ), Δ*htpG* (ΔG), and Δ*dnaKJ*Δ*htpG* (ΔKJG) *E. coli* strains, the physiological role of HtpG and its collaboration with DnaK and the DnaJ co-chaperone, in unstressed *E. coli* cells grown at 30°C. Confirming earlier findings (Calloni et al., 2012), we observed that in the double ΔKJ mutant, few polypeptides were mildly, albeit significantly less soluble than in WT, but that mass-wise, an important population of DnaK and DnaJ substrates, including many metabolic and respiratory enzymes, was largely reduced, likely by proteases. Unexpectedly, the decreased cellular levels of these aggregation-prone polypeptides were significantly less pronounced in the triple ΔKJG mutant, whose growth was also correspondingly improved at 37°C, compared to the double ΔKJ mutant. The data suggest that in unstressed bacteria, HtpG regulates the proper folding by DnaK and DnaJ of many polypeptides. However, when DnaK and DnaJ are inactive or transiently overwhelmed by misfolding polypeptides under heat stress, a strongly upregulated HtpG can promote an excessive and detrimental degradation of misfolding polypeptides, possibly by the HslUV protease. Contrary to DnaK–DnaJ–GrpE–ClpB chaperones that can disaggregate and refold proteins with compromised structures (Goloubinoff et al., 1999; Diamant et al., 2000), HtpG-driven proteolysis would be irreversible, and this could affect the cellular function of essential proteins, limiting bacterial growth and increasing its sensitivity to heat stress.

## Materials and Methods

### Bacterial Strains, Phages, and Culture Conditions

Genetic experiments were carried out in the *E. coli* in W3110 genetic background strain (Bachmann, 1972). The W3110 mutant derivatives Δ*dnaKdnaJ*::Kan^R^ and Δ*lon*::Kan^R^ (Sakr et al., 2010) have been previously described. The Δ*htpG*::Kan^R^, Δ*lon*::Kan^R^, Δ*clpP*::Kan^R^, and Δ*hslV*::Kan^R^ alleles were obtained from strains JWK0462, JWK0429, JWK0428, JWK0427, and JWK3903 (Keio collection). All mutations described in this study were moved to the appropriate genetic background by bacteriophage P1-mediated transduction. Bacteria were routinely grown in the Luria–Bertani (LB) medium supplemented when necessary with either kanamycin (50 μg/ml) or ampicillin (100 μg/ml). The construction of the *dnaK*-protease and *dnaK htpG* double mutants was performed as follows. The Kan^R^ cassettes from W3110 Δ*htpG*::Kan^R^, Δ*lon*::Kan^R^, Δ*clpP*::Kan^R^, and Δ*hslV*::Kan^R^ were first removed using plasmid pCP20 as described in Datsenko and Wanner (2000). The Δ*dnaKdnaJ*::Kan^R^ mutant allele was then introduced into the *htpG* and the various protease mutants, thus leading to strains W3110 Δ*htpG* Δ*dnaKdnaJ*::Kan^R^, Δ*lon* Δ*dnaKdnaJ*::Kan^R^, Δ*clpP* Δ*dnaKdnaJ*::Kan^R^, and Δ*hslV* Δ*dnaKdnaJ*::Kan^R^.

### Plasmid Construction

Plasmid pSE380Δ*Nco*I (Genevaux et al., 2004) has been previously described. To construct the high-copy number plasmid pSE-HtpG (pSE380 Δ*Nco*I-HtpG), the 1,875 bp *htpG* gene was PCR-amplified using primers HtpG-for (5′-CGGAATTCATGAAAGGACAAGAAACTCG-3′) and HtpG-rev (5′- CGAAGCTTTCAGGAAACCAGCAGCTGG-3′) using MG1655 genomic DNA as template. The PCR fragment was digested with *EcoR*I and *Hind*III and cloned into pSE380Δ*Nco*I previously digested with the same enzymes.

### Bacterial Viability Assays

Cultures of W3110 derivative strains were first grown overnight in LB medium at the permissive temperature (30°C for Δ*dnaKdnaJ*::Kan^R^ strains and 37°C for the other strains), diluted 1/50 into the same medium, further grown to mid-log phase, serially diluted 10-fold and spotted on the LB agar plates and incubated at the indicated temperatures. To monitor HtpG toxicity of the W3110 and its mutant derivatives, mid-log phase cultures of fresh transformants were grown at 30°C in LB-ampicillin medium containing 0.4% glucose, were serially diluted 10-fold and spotted on the LB ampicillin agar plates with or without isopropyl β-D-1-thiogalactopyranoside (IPTG; 1 mM), and incubated at 30°C.

### Sodium Dodecyl Sulfate Polyacrylamide Gel Electrophoresis/Western Blot

Western blots were performed as described in Bruel et al. (2012) and Angles et al. (2017). Proteins were separated by sodium dodecyl sulfate polyacrylamide gel electrophoresis (SDS-PAGE) and transferred onto a polyvinylidene fluoride (PVDF) membrane (Hybond-P, GE Healthcare, Chicago, IL, USA) using a semidry transfer system (Trans-Blot SD, Bio-Rad, Hercules, CA, USA) at 10 V for 30 min. Membranes were blocked for 1 h at room temperature (RT) or overnight at 4°C with 3% non-fat milk/Tris-buffered saline with Tween 20 (TBS–T; 50 mM Tris, 150 mM NaCl, pH 7.4, plus 0.05% Tween 20). A mouse antibody against DnaK (1/5,000 dilution) and a rabbit antibody against DnaK (1/5,000 dilution) were used as primary antibodies; and horseradish peroxidase (HRP)-conjugated rabbit immunoglobulin G (IgG) (1:5,000; Sigma-Aldrich, St. Louis, MO, USA) or mouse IgG (1:2,000; Sigma-Aldrich, St. Louis, MO, USA) were used as secondary antibodies. Membranes were developed using an enhanced chemiluminescence (ECL) Plus (GE Healthcare, Chicago, IL, USA) with a luminescence analyzer (LAS4000, Fujifilm VisualSonics Inc., Toronto, Canada).

### Proteomic Analysis

Cultures of W3110 *E. coli* were grown at 30°C in five biological replicates for each strain and harvested mid-log phase. Cells were lysed with lysozyme following resuspension. For the analysis of total (insoluble + soluble) protein content, lysed cells were adjusted to 8 M urea and a brief ultrasonication (3 × 10 s pulses) was performed to ensure complete protein solubilization. After a 2-h digestion at 37°C with Lys-C, the solution was diluted to adjust urea to 2 M, and then trypsin was added and incubated overnight at 37°C. For the analysis of insoluble protein fractions, insoluble proteins were first isolated by high-speed centrifugation (20,000 × g, 15 min, 4°C) following cell lysis. Pellets were then resuspended in 8 M urea and solubilized by brief ultrasonication (3 × 10 s pulses), and digestion was performed similarly to the total cell lysates. To maximize digestion yields from the insoluble protein fractions, a second round of trypsin digestion was performed, followed by another ~16-h incubation at 37°C.

In all cases, digests were desalted, resuspended in aqueous 2% acetonitrile + 0.05% trifluoroacetic acid. After loading onto a trapping microcolumn (Acclaim PepMap100 C18, 20 mm × 100 μm ID, 5 μm, Dionex, Sunnyvale, CA, USA), peptides were separated on a custom-packed nanocolumn (75 μm ID × 40 cm, 1.8 μm particles, Reprosil Pur, Dr. Maisch), with a flow rate of 250 nl/min and a gradient from 4 to 76% acetonitrile in water + 0.1% formic acid, over 140 min. Eluted peptides were analyzed on an Orbitrap Fusion Tribrid Mass Spectrometer (Thermo Fisher Scientific, Bremen, Germany) operated in data-dependent mode, controlled by Xcalibur software (version 3.0.63) (Thermo Fisher Scientific, Bremen, Germany). Full survey scans were performed at a 120,000 resolution, and a top speed precursor selection strategy was applied to maximize the acquisition of peptide tandem mass spectrometry (MS/MS) with a maximum cycle time of 3 s. Higher-energy collisional dissociation (HCD) fragmentation mode was used at a normalized collision energy of 32%, with a precursor isolation window of 1.6 m/z, and MS/MS was acquired in the ion trap. Peptides selected for MS/MS were excluded from further fragmentation during 60 s. The data collected by the MS were processed for protein identification and quantification using the MaxQuant version 1.5.3.30 (Computational Systems Biochemistry Research Group, Max Planck Institute of Biochemistry, Martinsried, Munich, Germany), using the Andromeda search engine set to search the UniProt database restricted to the *E. coli* (strain K12) proteome (UniProt proteome ID: UP000000625, number of sequences: 4,306). Trypsin (cleavage after K,R) was used as the enzyme definition, allowing two missed cleavages. Carbamidomethylation of cysteine was specified as a fixed modification, whereas N-terminal acetylation of protein and oxidation of methionine were specified as variable modifications. The MS proteomics data have been deposited to the ProteomeXchange Consortium via the PRIDE (Vizcaino et al., 2016) partner repository (EMBL-EBI, Cambridge, United Kingdom), and can be accessed at http://www.proteomexchange.org with the dataset identifier PXD010014.

All data postprocessing and statistical analyses were performed using the custom MATLAB scripts. Intensity-based absolute quantification (iBAQ) and label-free quantification (LFQ) data were used as the basis for quantification. Instead of only using raw iBAQ intensities, we took advantage of the additional normalization introduced by the LFQ method (Cox et al., 2014) to recalculate “normalized iBAQs” by dividing LFQ intensities by the number of theoretically observable tryptic peptides, as specified in the original iBAQ publication (Schwanhausser et al., 2011). Since normalized iBAQs are proportional to protein molar quantities, protein mass fractions were obtained as $f_{i}=\frac{I_{i}M_{i}}{\sum_{k}I_{k}M_{k}}\;$, where *I*_i_ is the normalized iBAQ intensity of protein i, *M*_i_ is its molecular weight, and the index *k* runs over all identified proteins. Then, the corresponding micromolar quantities *c*_i_ were derived using an estimated total intracellular protein concentration of *C*_T_ = 235 mg/ml (Zimmerman and Trach, 1991; Ellis, 2001): $c_{i}=\;10^{6}.C_{T}.\frac{I_{i}}{\sum_{k}I_{k}M_{k}}$.

An additional normalization was then performed to correlate protein abundances in insoluble fractions to those in the total protein fractions. A list of 281 *bona fide* membrane proteins was obtained from the Uniprot database. These known, fully insoluble proteins should therefore have the same mass fractions in the total and pellet mass fractions. For this equality to hold, log–log scatter plots of pellet vs. total mass fractions (**Figure 2A**) were used to perform curve fitting procedures (separately for each *E. coli* strain) of the form Log P = αLog T + β, where P and T correspond to pellet and total mass fractions, respectively. The obtained normalization coefficients α and β were then applied to the pellet mass factions of all identified proteins.

Statistical analyses using our five biological replicates were then performed, first to determine which proteins were significantly quantified (i.e., had a mass fraction significantly larger than zero). This was done using *t*-tests with a *post-hoc* Benjamini–Hochberg false discovery rate (FDR)-controlling procedure to account for multiple testing, at an FDR threshold of 0.01. Finally, significant differences in abundance or solubility between pairs of *E. coli* strains were determined using two-sample *t-*tests followed by the Benjamini–Hochberg procedures using an FDR cutoff of 0.05.

## Results

### Mutation in *htpG* Partially Suppresses the Growth Defect of the Double Δ*dnaKdnaJ* Mutant at 37°C

**Figure 1:**
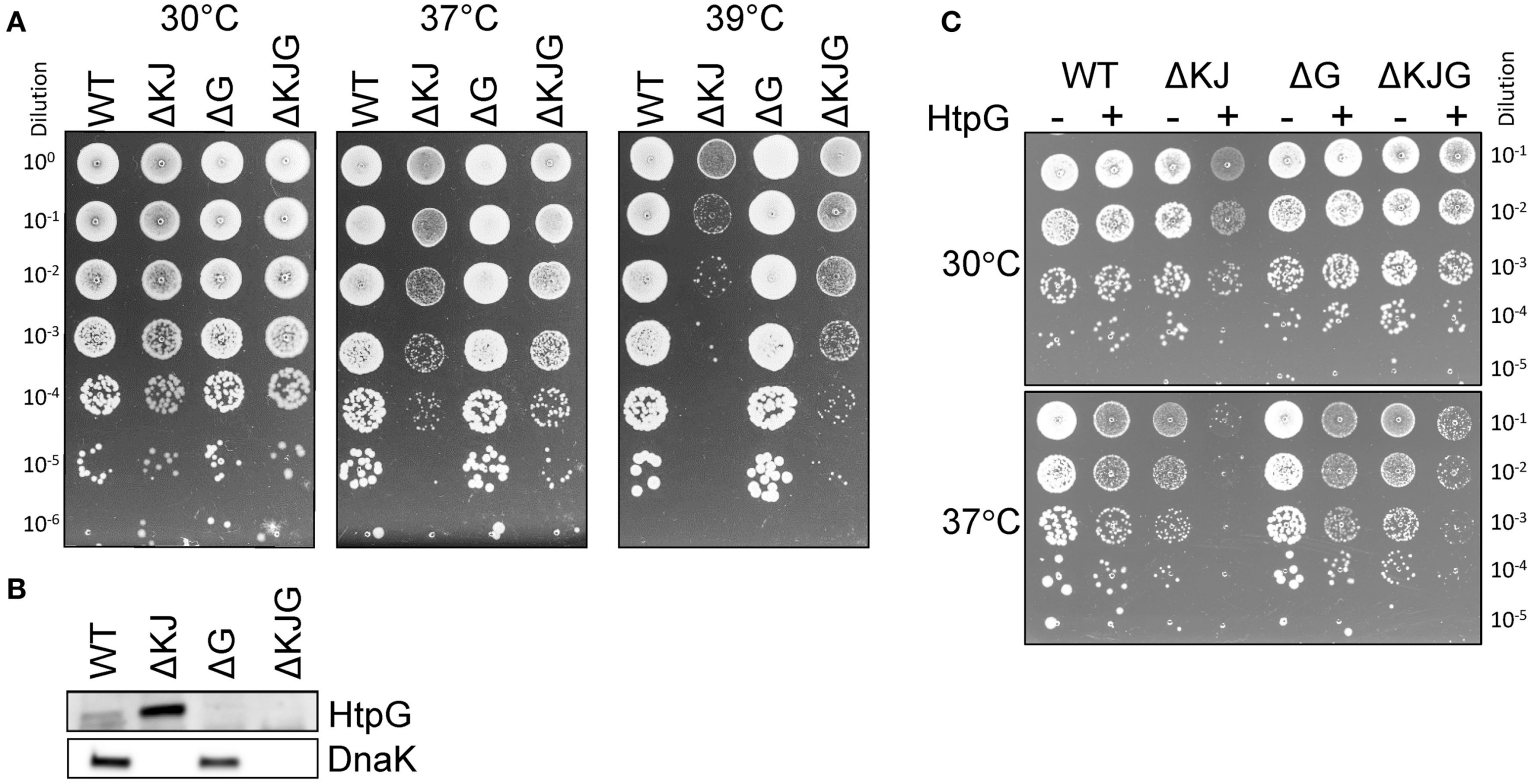
Mutation in htpG partially suppresses the growth defect of a ΔKJ mutant. **(A)** Mid-log phase cultures of wild-type (WT) W3110 *Escherichia coli* cells, ΔKJ, ΔG, and ΔKJG *E. coli* strains were serially diluted 10-fold and spotted on the Luria–Bertani (LB) agar plates and grown O/N at the indicated temperatures. **(B)** Western blots of whole cell extracts using anti-DnaK and anti-HtpG antibodies. **(C)** Toxicity of HtpG overexpression in ΔKJ background. Fresh transformants of W3110 derivative strains containing either the (empty) control plasmid (pSE380-LacZ) or HtpG (pSE380-htpG) were grown at 30°C, serially diluted 10-fold and spotted on the LB ampicillin agar plates supplemented with 1 mM IPTG for induction of HtpG overexpression. Plates were incubated for 1 day at 30°C (top) or 37°C (bottom).

In order to address a potential synergy between the DnaKJ chaperone machine and HtpG, we first designed a set of isogenic *E. coli* mutants in W3110 (WT) strain background, namely W3110 Δ*dnaKdnaJ* (ΔKJ) W3110 Δ*htpG* (ΔG) and W3110 Δ*dnaKdnaJhtpG* (ΔKJG) and tested their ability to grow at various temperatures. No major differences in growth were observed at 30°C. At higher temperatures, ΔKJ growth was severely affected, as initially shown by Bukau and Walker (1989) (Figure 1 and Supplementary Figure 1). Strikingly, we observed at 37°C and 39°C that the triple ΔKJG mutant grew significantly better than the ΔKJ mutant (Figure 1A), suggesting that the presence of endogenous HtpG was deleterious in a background lacking DnaK and DnaJ chaperones (Figure 1B). This harmful effect was confirmed by overexpressing HtpG from a plasmid, which dramatically inhibited the growth of ΔKJ but not of WT, already at 30°C (Figure 1C). In contrast, the overexpression of HtpG affected less on the growth of ΔKJG at 30°C (Figure 1C), possibly because of the complete absence of endogenous HtpG in this strain, contrary to ΔKJ where it is increased about 5-fold compared to WT (Supplementary Table 1).

We next used label-free MS-based absolute quantitative proteomic analysis (Cox et al., 2014), in an attempt to address and further identify particular *E. coli* polypeptides that specifically accumulate, degrade, and/or aggregate in cells lacking, either HtpG, DnaK/DnaJ, or all three. We identified and quantified the most abundant proteins in the total protein fractions of WT, ΔKJ, ΔG, and ΔKJG strains grown at 30°C and quantified their corresponding amounts in insoluble fractions. To optimize the statistical significance of the mean quantitative values obtained for each identified protein, we analyzed by MS five independent biological samples of total (soluble and insoluble) proteins and the five corresponding insoluble-only fractions for each of the four strains: WT, ΔKJ, ΔG, and ΔKJG, a total of 40 separately grown and independently prepared protein samples.

**Figure 2:**
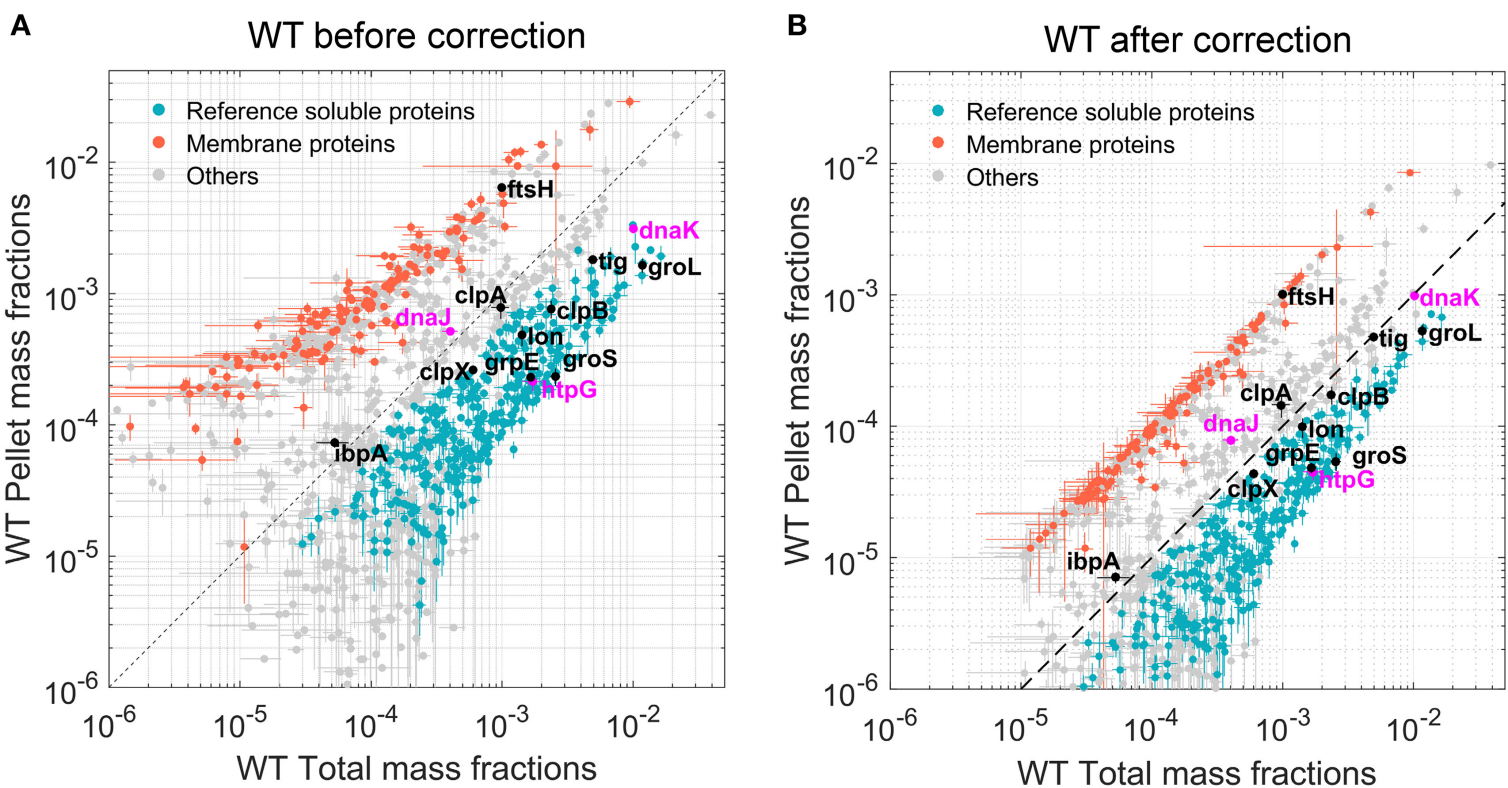
Quantitation of the most abundant soluble and insoluble proteins in wild-type (WT) *Escherichia coli* cells. Scatter plot of the relative mean mass fractions of 1,421 proteins from WT W3110 *E. coli* that were significantly quantified in the total fraction containing both soluble and insoluble proteins (X-axis), against only in the insoluble pellet fraction, containing mostly the insoluble proteins (Y-axis). **(A)** Before correction for the overrepresentation of the insoluble proteins in the pellet fractions. **(B)** After correction. Orange dots: 281 membrane proteins identified as such by the Uniprot database. Blue dots below the hatched line: proteins with a significant solubility index equal or >90%. Gray circles: other less significantly soluble proteins. Magenta dots: DnaK, DnaJ, and HtpG. Black dots, other canonical chaperones and major proteases.

The total protein fractions from the five biological samples of WT *E. coli* cells grown separately at 30°C were first analyzed. Quantification was based on LFQ intensities (Schwanhausser et al., 2011; Cox et al., 2014), which were first normalized into mass fractions (i.e., how much each protein contributes to the total protein mass per cell). We then took advantage of our five biological replicates to filter out proteins with very low average abundance and high variance *via t*-tests with multiple testing correction (see section “Materials and methods”). Of the estimated ~4,300 putative open reading frames of the *E. coli* genome (Kitagawa et al., 2005), 1,339 proteins with significant mean mass fraction values were identified and quantified (their FDR values were below 0.01, Supplementary Table 2). Although being only one-third of the total gene-encoding potential of the bacterial genome, these 1,339 most abundant proteins summed up to 97.2% of the total protein mass of the cell. A scatter plot of the individual mass fractions from these proteins (Figure 2A) showed that the pellet mass fractions of the 281 *bona fide* identified membrane proteins (Figure 2, orange dots) were systematically higher than their corresponding total mass fractions, and the sum of the total mass fractions of these 281 proteins represented 4.25% of the total protein mass (Supplementary Table 2); however, in the pellet fractions, they summed up to 28% of the WT *E. coli* pellet sample. This ~6.5-fold enrichment of pellet mass fractions compared to the true amounts in total cell lysates was confirmed and refined by a regression analysis of the total and pellet mass fractions of these membrane proteins. Based on the fitting performed on the 281 membrane proteins, all pellet mass fraction values were scaled down by a factor of ~6.5 for WT *E. coli*. The corrected scatter plot (Figure 2B) showed that the known membrane proteins then became positioned close to the 45° diagonal, which is the expected position of fully water-insoluble proteins, such as water-insoluble membrane-spanning and large insoluble structural proteins (Figure 2, orange dots). This procedure was then performed on the data from the other three *E. coli* strains, so as to perform meaningful comparisons between total and pellet mass fractions in each strain (Supplementary Table 3 and Supplementary Figure 3). The corrected plot provided a relative solubility index for each significantly quantified protein. Because of the relatively low degree of significance of these solubility index values, and for simplicity, we defined here as *fully soluble*, all proteins that were found to be at least 90% soluble (Figure 2, gray and blue dots, below the hatched line) and as the *fully insoluble*, all proteins that found in the plot to be <30% soluble (Figure 2, gray and orange dots, above the hatched line). To further validate this calculation, we verified that FtsH, a membrane-embedded essential protease, was located among the insoluble membrane proteins. In contrast, most other classical intracellular molecular chaperones and proteases, which are acknowledged soluble proteins, were above the 90% solubility threshold (Figure 2, black and blue dots). Noticeably, IbpA and DnaJ appeared slightly less soluble (Figure 2, gray dots), but given that IbpA can assemble into variably large oligomers (Kitagawa et al., 2002) and that both are known to associate with misfolded and insoluble aggregates, they might turn up being partly less soluble, even in WT cells grown at 30°C.

Taking an estimated total protein concentration in *E. coli* cells of 235 mg/ml (Zimmerman and Trach, 1991; Ellis, 2001) and knowing the specific molecular weight of each of the quantified polypeptides, we could translate the relative mean mass fraction values into cellular concentration estimates, expressed in μM (protomers). Thus, in WT cells grown at 30°C, the concentrations of DnaK, DnaJ, and HtpG were, respectively, 34.3, 2.3, and 5.6 μM protomers. There were 48 μM GroEL protomers and 58 μM GroES protomers (Supplementary Table 1), corresponding to about 3 μM of so-called footballs, which are functional GroEL_14_[GroES_7_]_2_ oligomers capped on both sides by GroES_7_ (Azem et al., 1995). Validating our methodological approach to convert LFQ MS data (Cox et al., 2014) into protein cellular concentrations, the concentrations deduced from the MS data showed a very good correlation with five previously published estimated concentrations of *E. coli* proteins obtained by similar and non-MS methods (Craig et al., 2004; Valgepea et al., 2010; Arike et al., 2012; Krug et al., 2013; Schmidt et al., 2016) (Supplementary Figure 2). We then applied the same statistical analysis to total and insoluble fractions of the ΔKJ, ΔG, and ΔKJG strains, which produced, respectively, 1,061, 1,156, and 1,056 significantly quantified proteins, summing up to be, respectively, 96.7, 97.3, and 96.7% of the total protein mass of the cells (Supplementary Figure 3).

### Analysis of Significant Differences in Protein Concentrations Between WT and Mutant Strains

**Figure 3:**
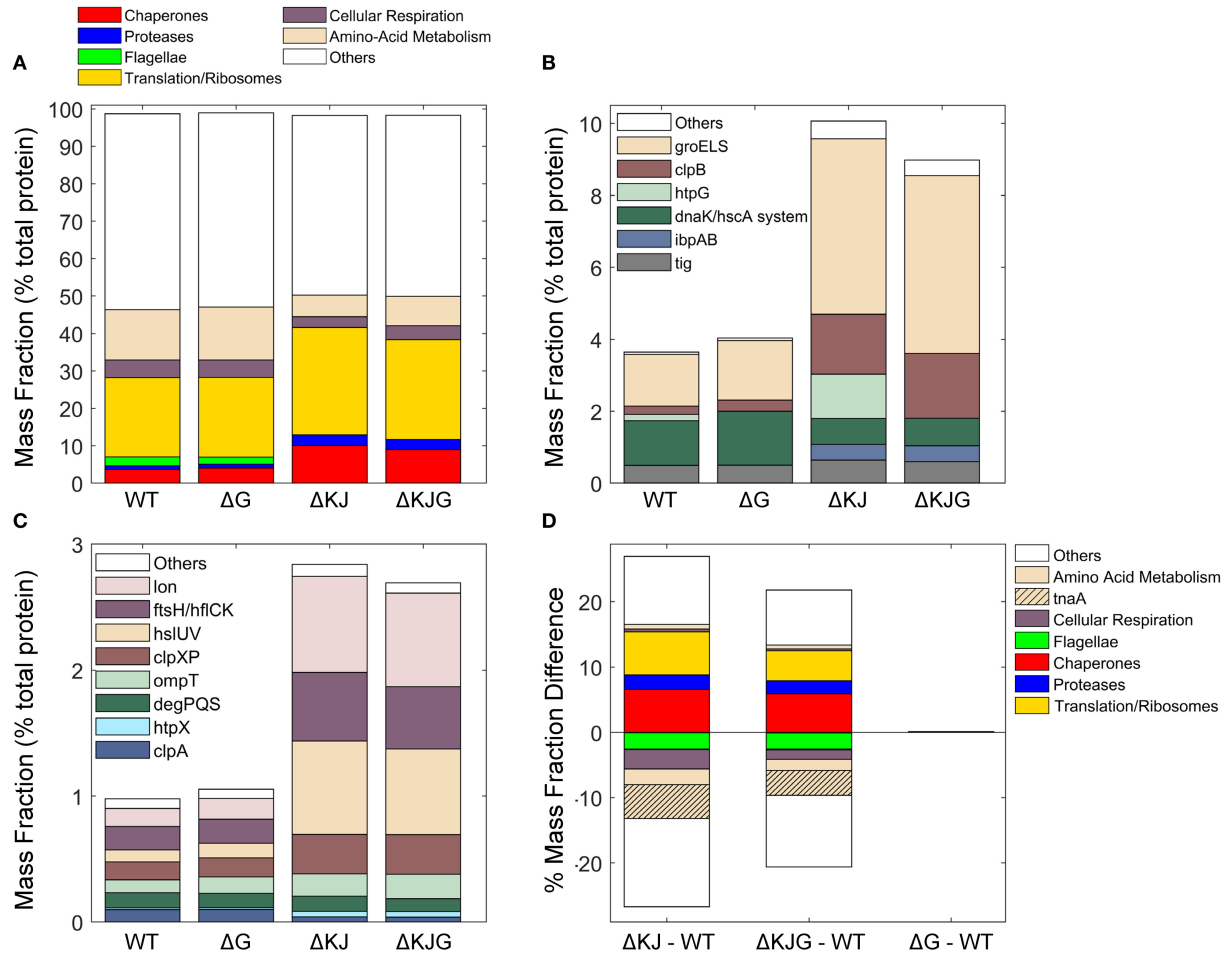
Analysis of different protein functional categories in the *Escherichia coli* variants. **(A)** Distribution of the principal functional categories of significantly quantified proteins in the four *E. coli* strains, Data are presented as mass fractions. Functional categories were manually defined according to both previous knowledge from the literature and Gene Ontology (GO) annotations. **(B)** Mass fractions of individual proteins belonging to the chaperone category. The dnaK/hscA system (dark green bar) comprises dnaK, dnaJ, grpE, cbpA, djlA, hscA, and hscB. The white bar (“Others”) comprises hslO, and spy. **(C)** Mass fractions of individual proteins belonging to the protease category. **(D)** Mass fraction differences (expressed in % of the total intracellular proteins) between wild type and the various deletion mutants. When absent in their respective mutants, the proteins DnaK, DnaJ, and HtpG were excluded from the analysis.

Our data provided precise protein concentrations with high statistical significance for WT and the three mutant strains. Compared to WT, the ΔKJ deletion caused a massive net significant accumulation of 635 proteins accounting for 26.9% of the total protein mass that was counterbalanced by net significant mass loss in 511 proteins, accounting for 26.7% of the total protein mass (Supplementary Table 2). Our data confirmed earlier quantitative proteomic data on ΔKJ by Calloni et al. (2012), showing with SILAC-based MS analysis, that in the absence of DnaK and DnaJ, there was a dramatic increase in the cellular concentrations of the other remaining molecular chaperones and proteases. We found that the whole chaperone load of ΔKJ cells was increased from 3.2 to 8.8%, despite the loss of DnaK and DnaJ that accounted in WT cells for ~1% of the total protein mass (Figures 3A,B and Supplementary Table 2). A similar increase of the chaperone load was observed in the triple mutant ΔKJG, indicating that HtpG is not involved in the degradation of the σ^32^ transcription factor, at variance with DnaK and DnaJ (Lim et al., 2013). A close-up analysis of the bacterial “chaperome” (Figure 3B and Supplementary Table 1) showed that the complete deletion of *dnaK* and *dnaJ* in ΔKJ was counterbalanced by a 3-fold increase in GroEL and GroES, and a 7-fold increase in HtpG and ClpB (Supplementary Table 1). The small Hsps, IbpA, and IbpB, which were virtually undetected in WT, were massively accumulated in ΔKJ (54- and ~1,400-folds for IbpA and IbpB, respectively) and in ΔKJG mutants, but not in ΔG. Noticeably, large fold-change values did not necessarily translate into extensive variations of protein abundances. Thus, although IbpB was upregulated ~1,400-fold in ΔKJ, it merely contributed a net 0.2% increase to the total protein mass of the cell. In contrast, GroEL, which was upregulated merely 3.4-folds, contributed a considerable net 2.4% increase to the total protein mass of the cell, i.e., 12 times more than IbpB.

Similar to classical chaperones, but mass-wise to a lesser extent, ATP-dependent proteases, such as Lon, FtsH, ClpXP, and HslUV also accumulated between 3- and 9-fold in ΔKJ and ΔKJG, but not in ΔG (Figure 3C and Supplementary Table 1). Only ClpA, which like ClpX associates to the ClpP protease, was less expressed in ΔKJ, in agreement with ClpA expression not being under the control of σ^32^ (Katayama et al., 1988) and with ClpXP, Lon, FtsH, and/or HslUV likely being involved in the degradation of proteins who failed to properly fold in the ΔKJ mutants. Noticeably, as previously shown by Calloni et al. (2012), DegPQS, which are non-ATPase periplasmic proteases and therefore are not expected to be involved in the degradation of cytosolic proteins, were less expressed in ΔKJ than in WT.

Both in ΔKJ and ΔKJG strains, there was also a marked increase of ribosomal proteins and of associated components of the protein translation machinery, as compared to WT; their proportion massively increased from 19.9% of the total mass in WT, to 27.2% in ΔKJ, and to 25.1% in ΔKJG. Because in ΔKJ, the amount of insoluble proteins at 30°C was not markedly higher than in WT, this implies that the mass gain from the newly synthesized chaperones, proteases, and ribosomes must have been counterbalanced by a corresponding mass loss from the synthesis-arrest and/or from the specific degradation by proteases of other abundant proteins, such as enzymes from the amino acid metabolism. This is in agreement with the major role that was initially found for DnaK in central metabolism (Angles et al., 2017) by microarray work (Fan et al., 2016), and with the earlier SILAC-MS proteomic observations that many DnaK–DnaJ-dependent substrates become significantly degraded in ΔKJ as compared to WT cells (Calloni et al., 2012).

We found that the remarkable high levels of metabolic enzyme tryptophanase (TnaA) in WT were strongly decreased from 6.4 to 0.9% of the total protein mass (a ~86% reduction) in ΔKJ. This result indicates that in the exponential phase of growth, TnaA cannot reach its native state without the assistance of DnaK and DnaJ and instead becomes targeted by the ~8-fold higher levels of HtpG to degradation by proteases, which are higher than twice more abundant. Other abundant proteins, such as the known DnaK interactors PflB and PutA (Calloni et al., 2012) (Supplementary Table 2) were also decreased in ΔKJ. In addition, confirming early reports that flagellum synthesis strictly depends on DnaK (Shi et al., 1992), we found that several members of flagellar machinery proteins, principally flagellin (FliC), were completely absent in ΔKJ. Interestingly, already at this coarse level of analysis of customized and Gene Ontology (GO) protein categories (Figure 3A), we observed almost no differences in the mass profile of the proteins in ΔG as compared to the WT strain. The only exception was ClpB, which out of ~1,300 significantly quantified proteins in both WT and ΔG strains was mildly more abundant in ΔG. The lack of proteomic phenotype in unstressed ΔG cells was confirmed by the triple mutant ΔKJG, which showed a protein profile quite similar to that of the double ΔKJ mutant. Yet, the extent of mass loss in enzymes involved in amino acid metabolism and in cellular respiration was found to be systematically less pronounced in ΔKJG (Figure 3D). This suggests a correlation between growth-slowing and the relative mass loss of key metabolic enzymes (Angles et al., 2017) in the ΔKJ mutant as compared to ΔKJG.

**Figure 4:**
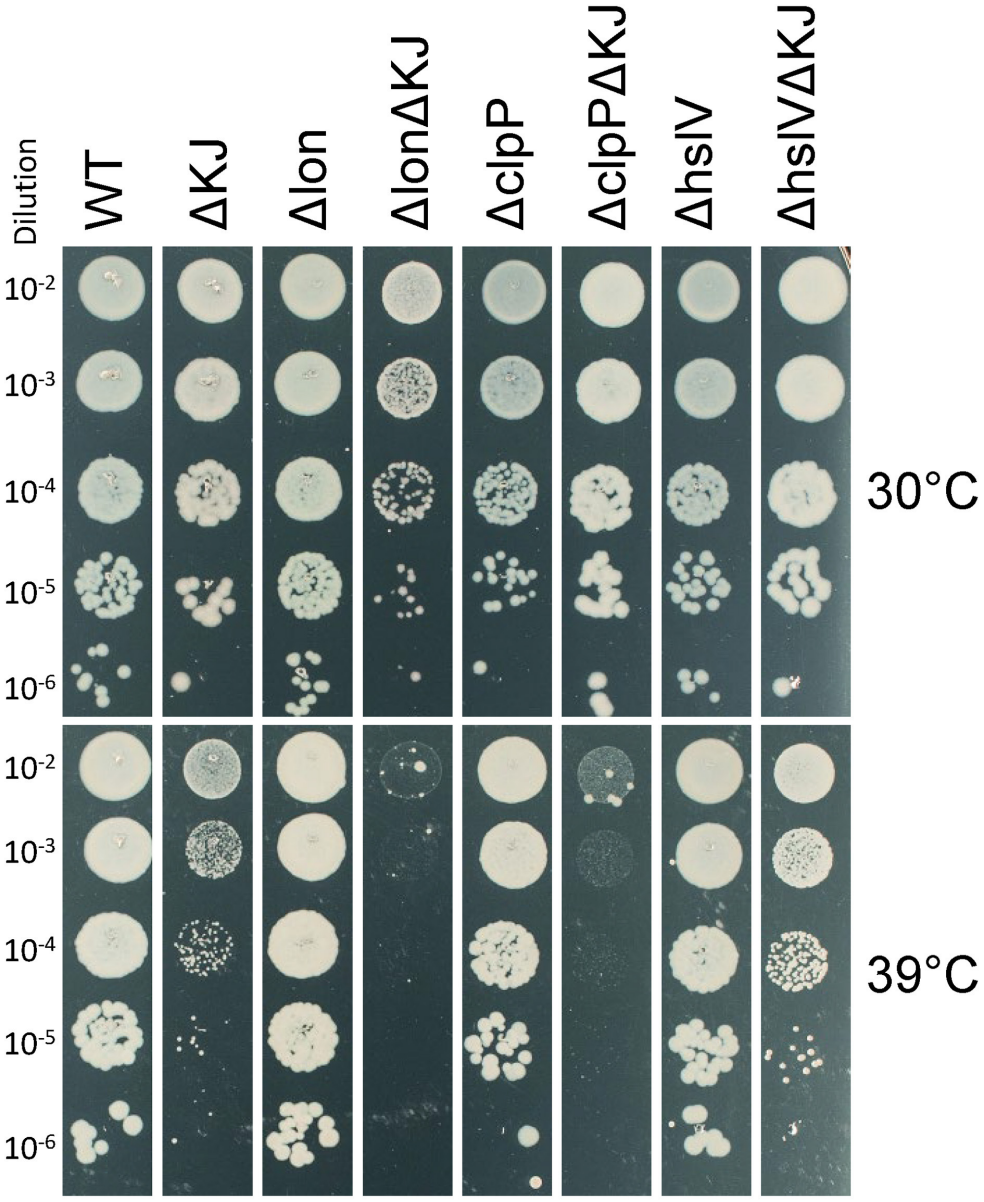
*hslV* deletion improves growth in the ΔKJ background. Serially 10-fold diluted cultures of wild type (WT), ΔKJ, or ΔKJ combined with the indicated single-protease deletions (Δ*lon*, Δ*clpP*, and Δ*hslV*) were spotted on agar plates and grown overnight at 30°C **(top)** or 39°C **(bottom)**.

We next addressed genetically, which of the main *E. coli* proteases might be involved in the HtpG-promoted degradation of misfolded DnaKJ substrates. The triple ΔKJ-protease mutants Δ*dnaKdnaJ*Δ*lon*, Δ*dnaKdnaJ*Δ*hslV*, and Δ*dnaKdnaJ*Δ*clpP* were grown at different temperatures. At 30°C, the deletion of *lon* impaired the growth of the ΔKJ strain (Figure 4). At 39°C, the deletion of *lon* or *clpP* further impaired ΔKJ growth, whereas, remarkably, the deletion of *hslV* partially suppressed the growth defect of the ΔKJ strain (Figure 4). Together with the results from the pulse-chase SILAC-MS experiments from Calloni et al. (2012), who showed increased protein degradation in ΔKJ compared to WT, and the pronounced reduction of TnaA in the ΔKJ strain (**Figure 6** and Supplementary Table 2), our finding showed that the HslV deletion improved growth of ΔKJ, suggesting that HtpG is involved in mediating the degradation, possibly *via* the HslUV protease, of aggregation-prone polypeptides that need DnaK–DnaJ to properly fold. The 935 proteins that significantly differed in ΔKJ from WT and the 834 proteins that significantly differed in ΔKJG from WT were next sorted according to protein categories. In both groups, chaperones, proteases, and proteins of the translation machinery were almost exclusively upregulated, whereas flagellar proteins, amino acid metabolic enzymes, and proteins of the energy metabolism (cellular respiration) were mostly downregulated (or degraded).

It was initially shown that compared to WT cells, ΔK cells massively accumulate insoluble proteins at 42°C, but not at 30°C (Mogk et al., 1999). Here, we found that in WT, ΔKJ, and ΔKJG strains grown at 30°C, the sum of the insoluble proteins was, respectively, 18.5, 20.5, and 18.9% of the total cellular proteins, suggesting that at 30°C the ΔKJ background contained slightly but more dramatically insoluble aggregates than the WT. Solubility differences were then assessed in a more stringent list obtained with *t*-tests (test with multiple testing correction, FDR cutoff 0.05) to determine which proteins were statistically differing in their solubility between WT and ΔKJ. The list was furthermore restricted to proteins with more than 10% point difference in solubility {i.e., |Solubility_WT_ – Solubility_Δ*KJ*_| > 0.1 [where Solubility = (1 – Pellet)/Total] because smaller solubility differences are unlikely to hold biological significance}. We thus obtained a list of 161 proteins (Supplementary Table 4), 133 of which were less soluble in ΔKJ than in WT. These 133 proteins, which summed up to 7.3, 10.4, and 9.8% of the total protein mass in WT, ΔKJ, and ΔKJG, respectively. Their average solubility decreased from 91% in WT to 70% in ΔKJ. The solubility of these proteins did not change in ΔKJG, being on average 71% as in ΔKJ. The proteomic analysis of the ΔKJ mutant thus indicated that, expectedly, ΔKJ contained somewhat less soluble proteins, though in relatively small numbers and contribution to the total proteome, which is consistent with the observation that at 30°C, the ΔKJ cells grew similarly to WT cells.

**Figure 5:**
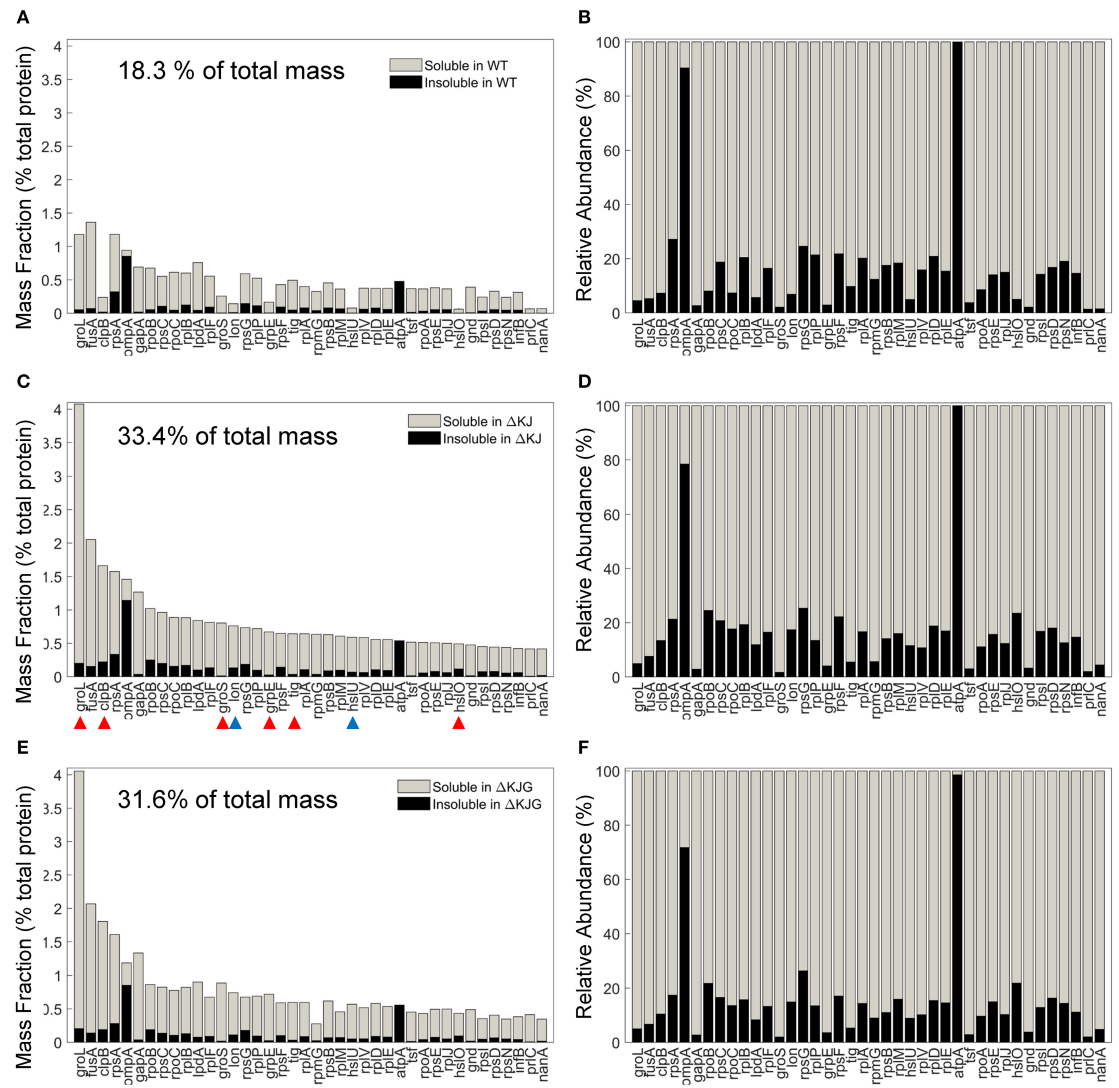
Mild aggregation of the most abundant proteins significantly upregulated in ΔKJ. **(A)** Absolute abundances (mass fractions of the total cellular protein amount) in wild type (WT) soluble (gray bars) and insoluble (black bars) fractions for the 40 most abundant proteins of ΔKJ *Escherichia coli* that were significantly upregulated in ΔKJ compared to WT. **(B)** Normalized abundances in WT *E. coli* of the same proteins as in **(A)**. Red arrowheads: chaperones; blue arrowheads: proteases. **(C)** Absolute abundances (mass fractions) of the same proteins as in **(A)**, but showing their amounts in ΔKJ soluble (gray bars) and insoluble (black bars) fractions **(D)** Same as in **(C)**, with normalized abundances. **(E)** Absolute abundances (mass fractions) of the same proteins as in **(A)**, but showing their amounts in ΔKJG soluble (gray bars) and insoluble (black bars) fractions. **(F)** Same as in **(E)**, with normalized abundances.

Chaperones are classically described as molecules that prevent protein aggregation. We therefore next compared the effects of the HtpG overexpression (in the ΔKJ strain), or its deletion into the ΔKJ background, on the specific solubility indexes of key individual proteins and their total cellular amounts. The 40 most abundant proteins that most significantly accumulated in ΔKJ compared to WT (Figure 5A) were either ribosomal proteins, chaperones (Figure 5C, red arrowheads), or proteases (Figure 5C, blue arrowheads). However, together they summed up to be 18.3% of the total protein mass of WT cells, they were nearly doubled (33.4%) in ΔKJ cells, and slightly less (31.6%) in ΔKJG cells (Figure 5E). Remarkably, although they were significantly less abundant in WT and ΔKJG than in ΔKJ, they all maintained the same degree of relative solubility (Figures 5B,D,F). Thus, the ΔKJ phenotype cannot be attributed to a decrease in the solubility of the proteins that became most upregulated in ΔKJ.

**Figure 6:**
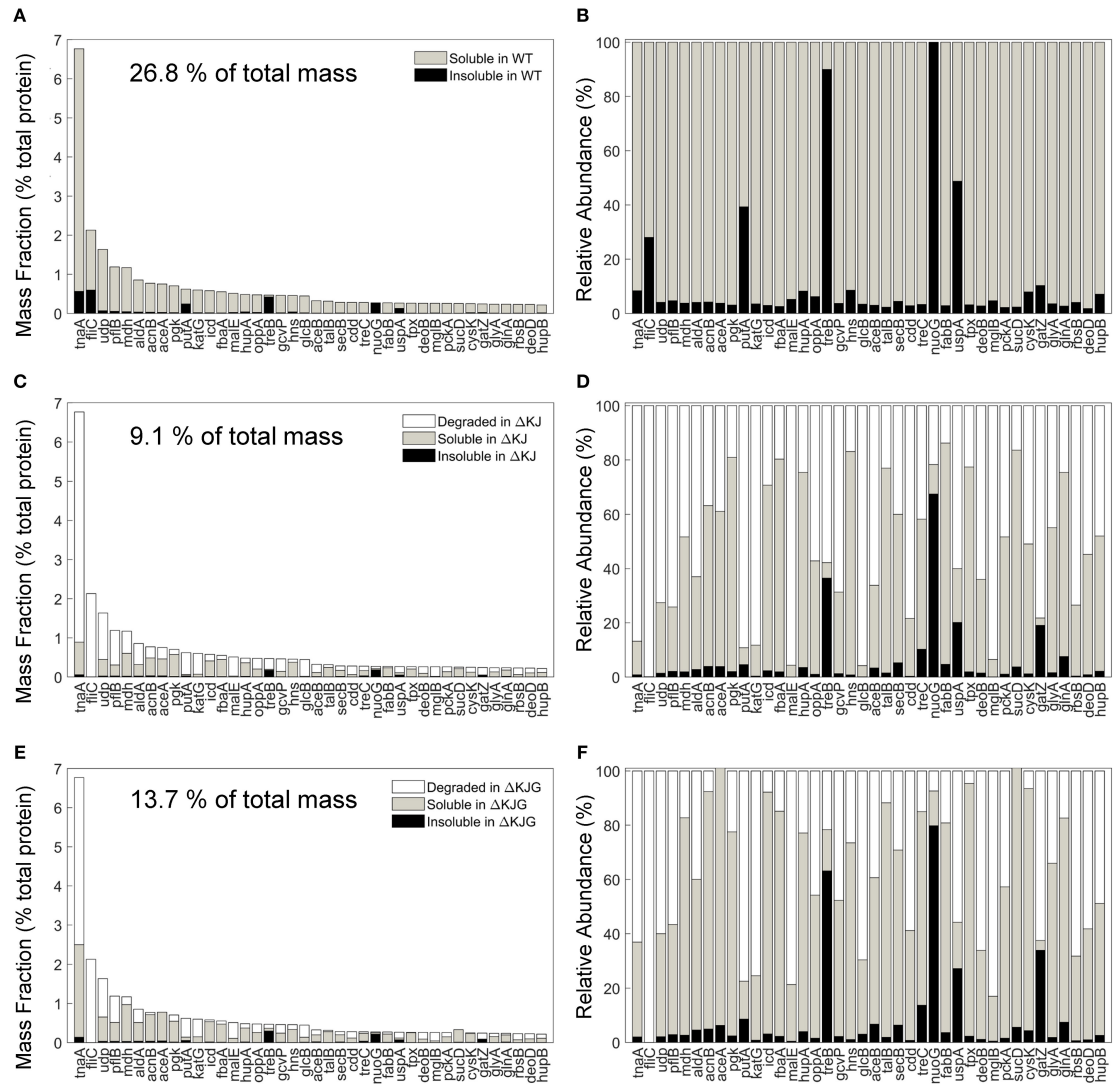
Protein degradation is a prevalent phenotype in ΔKJ and partially rescued in ΔKJG. **(A)** Absolute abundances (mass fractions of the total cellular protein amount) in wild-type (WT) soluble and insoluble fractions for the 40 most abundant proteins of WT *Escherichia coli* that were downregulated in ΔKJ. **(B)** Normalized abundances in WT *E. coli* of the same proteins as in **(A)**. **(C)** Absolute abundances of the same proteins as in **(A)**, but with a partitioning showing the amounts of each protein degraded in ΔKJ (white bars); the soluble (gray bars) and insoluble (black bars) amounts in. **(D)** Same as in **(C)**, with normalized abundances. **(E)** Same absolute abundances as in **(A,C)**, but partitioned in order to show the quantities degraded in ΔKJG with respect to WT (white bars); the soluble (gray bars) and insoluble (black bars) amounts in ΔKJG. **(F)** Same as in **(E)**, with normalized abundances.

The involvement of molecular chaperones, DnaK in particular, in intracellular protein degradation of proteins has long been documented (Sherman and Goldberg, 1996) and recently confirmed in greater detail by SILAC-MS with pulse-chase experiments (Calloni et al., 2012). We therefore next identified the 40 most abundant proteins in WT (Figures 6A,B) that were significantly less abundant in ΔKJ (Figures 6C,D) and found that most were enzymes involved in cellular respiration and in the amino acid metabolism, in particular TnaA. Together, the mass of these 40 most reduced proteins in ΔKJ compared to WT was 26.8% in WT, 9.1% in ΔKJ, and 13.7% in ΔKJG, corresponding to a dramatic 3-fold mass loss in ΔKJ, compared to WT, which was lessened to a 2-fold in ΔKJG. Indeed, 39 of these 40 proteins were found to be significantly more abundant in ΔKJG (Figures 6E,F) than in ΔKJ (Figures 6C,D), further suggesting that the deletion of *htpG* leads to less degradation of these proteins that otherwise tend to misfold in the ΔKJ background.

## Discussion

The Hsp90s are members of a highly conserved family of ATP-hydrolyzing molecular chaperones, which together with the Hsp70s form the core of the cellular protein quality control machinery (Taipale et al., 2010; Balchin et al., 2016). The various cytosolic and organellar orthologs of Hsp90 are among the most abundant proteins of animal cells, reaching ~1% of the total protein mass (Priya et al., 2013; Gat-Yablonski et al., 2016). Here, we find that in bacteria, DnaK levels are also ~1% as in the cytosol, but that HtpG levels are about seven times lower, unless DnaK is deleted (Supplementary Figure 4). Experimental evidence suggests that HtpG functions in close collaboration with DnaK. Using *in vitro* assays with purified chaperones, Genest et al. (2011) showed that when assisted by GrpE and especially by CbpA, which is a bacterial type B J-domain-containing Hsp40, HtpG and DnaK collaborate at converting artificially unfolded or misfolded polypeptides into native proteins in an ATP hydrolysis-dependent manner. Moreover, this cooperative activity involves the direct interaction between DnaK and HtpG (Nakamoto et al., 2014; Genest et al., 2015), with the DnaK–HtpG interface being localized to the DnaJ-binding site within DnaK (Kravats et al., 2017). In the case of denatured luciferase refolding, there is evidence that the direct interaction between DnaK and HtpG promotes the refolding of misfolded clients through an ATP-dependent sequential mechanism requiring the transfer of folding intermediates of decreasing exposed surface hydrophobicity from DnaK to HtpG for complete refolding to occur (Genest et al., 2018; Moran Luengo et al., 2018). Moreover, the collaboration between Hsp70 and Hsp90 has also been demonstrated in other organisms, such as yeast (Genest et al., 2013) and metazoans (Genest et al., 2018; Moran Luengo et al., 2018), where Hsp70/Hsp90 interactions are key to the maturation of the steroid hormone receptor (Li et al., 2012).

Relatively little is known about the precise molecular mechanism by which Hsp90s carry their specific ATP-fueled protein-restructuring functions. It is not known why are they so evolutionarily conserved, and what makes them particularly essential under both physiological and stress conditions, compared to other types of molecular chaperones. Similar to Hsp60s (GroEL), Hsp70s (DnaK), Hsp40s (DnaJ), and small Hsps (IbpA and IbpB), Hsp90s (HtpG) (*E. coli* chaperones in brackets) can bind misfolding polypeptides and prevent the formation of inactive and potentially toxic protein aggregates (Stefani and Dobson, 2003). However, Hsp60/10, Hsp70/40, and Hsp104/70 are unfolding enzymes that can use the energy of ATP hydrolysis to forcefully translocate polypeptide to convert stable misfolded or oligomeric protein complexes into native proteins, and very little is known about the particular nature of the structural changes applied by the Hsp90s onto their bound protein clients (Finka et al., 2016). Hsp90 assembles into very dynamic dimers (Shiau et al., 2006; Krukenberg et al., 2011; Li et al., 2012; Flynn et al., 2015; Mayer and Le Breton, 2015). There are profound structural differences between the apo-, ATP-, and ADP-bound states. A central question about the chaperone mechanism of Hsp90s is the role of specific co-chaperones. In the cytosol of eukaryotes, Hop, Aha1, and P23 are three conserved Hsp90 co-chaperones controlling precise structural changes in the Hsp90 dimer during the various stages of the ATPase cycle (Mayer and Le Breton, 2015). Yet, there are no apparent orthologs of Hop, Aha1, and P23 in the lumen of the endoplasmic reticulum, in the stroma of mitochondria, or in the cytosol of eubacteria, to co-chaperone, respectively, GRP94, Trap1, and HtpG. In contrast, Hsp70 is always coexpressed with Hsp90s in all the ATP-containing compartments of cells, raising the possibility that Hsp70 is acting as the most conserved co-chaperone to all prokaryotic, organellar, and eukaryotic forms of Hsp90s (Finka et al., 2011). There are numerous examples of Hsp90 and Hsp70 acting together as in the case of steroid hormone receptor activation (Echeverria and Picard, 2010) and in a context devoid of a Hop co-chaperone as in *E. coli* cells (Nakamoto et al., 2014). *In vitro*, HtpG from *E. coli* and cyanobacteria seem to collaborate with the DnaK chaperone system to remodel denatured proteins (Genest et al., 2011; Nakamoto et al., 2014). However, owing to the early finding of specific inhibitors, the involvement of Hsp90s in cellular signaling and stress protection has been demonstrated in eukaryotes, and very little is known about the role of Hsp90s in proteobacteria, where it is apparently not essential.

In *E. coli*, the deletion of the *htpG* gene, though highly conserved, leads only to a very minor phenotype observable only under elevated temperatures (Bardwell and Craig, 1988; Thomas and Baneyx, 2000; Grudniak et al., 2013; Press et al., 2013). In cyanobacteria, it is involved in phycobilisome assembly (Motojima-Miyazaki et al., 2010; Sato et al., 2010; Press et al., 2013), in heat stress (Thomas and Baneyx, 1998, 2000), and it participates in oxidative stress resistance (Tanaka and Nakamoto, 1999; Hossain and Nakamoto, 2003). In pathogenic eubacteria, HtpG may be involved in bacterial immunity (Yosef et al., 2011) and virulence associated with toxin synthesis (Vivien et al., 2005; Dang et al., 2011; Verbrugghe et al., 2015; Garcie et al., 2016). Honore et al. (2017) recently found that HtpG from *S. oneidensis* is essential for the growth at high temperature and identified TilS, an essential protein that controls the production of the rare AUA-Ile tRNA, as an HtpG client (Honore et al., 2019); however, in the case of TilS, the protein is stabilized by HtpG under heat stress (Honore et al., 2017). HtpG is also needed for biofilm formation and secretion of certain enzymes, such as β-lactamase in *E. coli* (Grudniak et al., 2013). Moreover, a recent genome-wide co-evolution study in *E. coli* predicted that under physiological conditions, HtpG may assist the folding of chemotaxis-related proteins, such as the chemoreceptor kinase CheA. HtpG was reported to be essential only in particular cases, such as for the activity of the CRISPR/Cas system (Yosef et al., 2011). Yet, the precise mechanism of action of the bacterial Hsp90, the identity of its substrates, and its role under physiological and stress conditions remains elusive.

Here, we found that in unstressed *E. coli* cells at 30°C, the amount of HtpG was in general less abundant than in animal cells, only 0.15% of the total proteins, but that in ΔKJ cells, it was increased to 1%. Rationalizing their traditional, albeit inaccurate, designation as Hsps, this amount may increase by ~10% following a heat shock, such as a fever (Finka et al., 2011, 2015). Both Hsp90 and Hsp70 molecules can passively bind limited amounts of stress-labile polypeptides, thereby preventing some aggregations, albeit only to the limited extent of the individual binding (or so-called holdase) capacity of each chaperone (Wiech et al., 1992; Street et al., 2014; Kravats et al., 2017). Importantly, in the presence of an excess of misfolding polypeptides, Hsp70, Hsp100s, and possibly Hsp90 too can use the energy of ATP hydrolysis to unfold stably misfolded polypeptides and catalytically convert them into native proteins, even under stressful conditions that disfavor the native state (Genest et al., 2015; Finka et al., 2016; Goloubinoff et al., 2018). As in the case of specific Hsp70s, elevated cellular levels of Hsp90 are hallmarks of various types of stresses (Priya et al., 2013; Finka et al., 2015). At 41°C, human cell cultures can accumulate 50% more Hsp90s, compared to 37°C (Finka et al., 2015). Likewise, when naive rat liver cells at 37°C are being stressed by chronic excessive (*ad libitum*, AL) food intake (Supplementary Figure 4, AL), the balance between Hsp70s and Hsp90s is disrupted, with liver cells in AL expressing ~50% more Hsp70s than in calorie-restricted (RES) rat liver cells, whereas Hsp90s are decreased in AL compared to RES, therefore altering the stoichiometry between Hsp70s and Hsp90s (Gat-Yablonski et al., 2016). Justifying that Hsp90s and Hsp70s should be major drug targets for cancer therapy, various immortalized cancer lines constitutively overexpress Hsp90s and Hsp70s at 37°C. Together, they can prevent the oligomerization and the activation of various client proteins, such as HSF1 (Abravaya et al., 1992), IκB (Weiss et al., 2007), and block caspase activation (Multhoff, 1997; Jaattela et al., 1998; Lanneau et al., 2007; Neckers et al., 2007; Vartholomaiou et al., 2016), thereby promoting immortality of cancer cells and the general resistance to physical stresses and chemotherapeutic agents.

We found that under non-stressful growth conditions (30°C), many proteins, in particular belonging to amino acid metabolism and cellular respiration categories, were significantly less abundant in DnaK/J-knockout cells, a phenotype that was mostly reversed by the further deletion of HtpG. Although a lower replenishment rate could account for this observation, an increased abundance of ribosomal and translation-related proteins in the ΔKJ cells, and in line with previous pulse-chase SILAC-MS experiments (Calloni et al., 2012), a higher degradation of these proteins in ΔKJ cells is the most likely explanation.

Thus, the data suggest that bacterial Hsp90 is involved in the sorting of aggregation-prone proteins toward native refolding by the Hsp70/Hsp40 system, or in case of Hsp70/Hsp40 failure, toward more degradation, for example through the HslUV protease. Such interplay between chaperones and proteases has been observed in *E. coli*, where trigger factor (TF) specifically cooperates with the ClpXP protease to promote the degradation of specific proteins, which represent a significant portion (about 2%) of newly synthesized proteins (Rizzolo et al., 2021). Accordingly, it has been demonstrated that in the α-proteobacterium *Caulobacter crescentus*, DnaK depletion is suppressed by HslUV, by attenuating the activity of the σ^32^ factor (Schramm et al., 2017). The interplay between HtpG and the HslUV protease has been further confirmed in *S. oneidensis*, where the deleterious Δ*hsp90* phenotype was shown to be compensated by the further deletion of *hslUV* in a Δ*hsp90* background (Honore et al., 2019).

Our data thus suggest a role for HtpG in the bacterial proteostasis network: it could identify proteins that fail to properly fold when DnaK is absent or overwhelmed by stress-induced aggregation. Even without stress, some nascent polypeptides can fold spontaneously to the native state, whereas others are known to necessitate TF (Deuerling and Bukau, 2004) (Supplementary Figure 5). Others that misfold, for example, in TF mutants may need to be actively unfolded by bacterial Hsp70s and Hsp40s, in order for them to reengage onto the native folding pathway to the functional state. Failing that in the ΔKJ knockout strain, where TF is not dramatically more abundant, but where HtpG is seven times more abundant, misfolding proteins are redirected by HtpG to be degraded preferably by HslV and HslU being, respectively, 9 and 7.5 times more abundant in ΔKJ than in the WT (Supplementary Table 1). In *S. oneidensis*, Hsp90 prevents the essential protein TilS from HslUV-mediated degradation under non-stressful growth conditions where TilS is in its native state (Honore et al., 2019), thus suggesting that Hsp90 distinguishes between native and non-native sates and interacts with downstream proteases accordingly.

A protein that has been already degraded has lost the possibility to be instead solubilized and reactivated by molecular chaperones. To carry its biological function, it must first be resynthetized, a process estimated to be 100–1,000 times more ATP consuming than the low-cost protein “repair” mechanisms of disaggregation and refolding to the native state mediated by DnaK, DnaJ, and GrpE (Sharma et al., 2011). The impaired growth of ΔKJ at 37°C could thus be caused by an excessive degradation of essential metabolic and respiratory proteins. Confirming that, the deletion of the *htpG* gene in ΔKJG (or of the *hslV* gene in ΔKJ) reduced protein degradation and partially restored bacterial growth at 37°C.

The artificial overexpression of Hsp90 in *Caenorhabditis elegans* embryos was recently shown to cause the massive degradation of muscle proteins (Bar-Lavan et al., 2016), indicating that in the cytosol of stressed eukaryotic cells, Hsp90 might also mediate the specific degradation of misfolding proteins that escaped the action of other members of the chaperone network. It will now be interesting to address which are the particular protein partners in bacteria that mediate the interaction of Hsp90 and Hsp70 clients with the cellular proteases and further address the possibility that also in eukaryotes, Hsp90s could mediate the degradation by proteasome of Hsp70 clients that failed proper folding under stress or owing to mutations.

## Data Availability Statement

The datasets presented in this study can be found in online repositories. The names of the repository/repositories and accession number(s) can be found in the article/Supplementary Material.

## Author Contributions

PGe, MQ, and PGo designed the experiments. BF, AF, M-PC-C, A-MC, and MQ performed the experiments and analyzed the results. PGe, PGo, BF, and AF wrote the manuscript. All authors contributed to the article and approved the submitted version.

## Conflict of Interest

The authors declare that the research was conducted in the absence of any commercial or financial relationships that could be construed as a potential conflict of interest.
