# Supplementary material for: Bacterial Hsp90 Facilitates the Degradation of Aggregation-Prone Hsp70–Hsp40 Substrates
Source: Front Mol Biosci. 2021 Apr 15;8:653073. doi: 10.3389/fmolb.2021.653073 (PMC8082187; doi:10.3389/fmolb.2021.653073)
Supplement: Supplementary file 1 [file Data_Sheet_1.pdf]

Supplementary Material for

Bacterial Hsp90 facilitates the degradation of aggregation-prone Hsp70-Hsp40 substrates

Bruno Fauvet[a], Andrija Finka[b], Marie-Pierre Castanié-Cornet[c], Anne-Marie Cirinesi[c], Pierre Genevaux[c], Manfredo Quadroni[d], and Pierre Goloubinoff[a]#

[a] Department of Plant Molecular Biology (DBMV), University of Lausanne, Switzerland.

[b] Department of Ecology, Agronomy and Aquaculture, University of Zadar, Croatia.

[c] Laboratoire de Microbiologie et de Génétique Moléculaires, Centre de Biologie Intégrative, CNRS, Université de Toulouse, France.

[d] Protein Analysis Facility, University of Lausanne, Switzerland.

#To whom correspondence should be addressed: pierre.goloubinoff@unil.ch

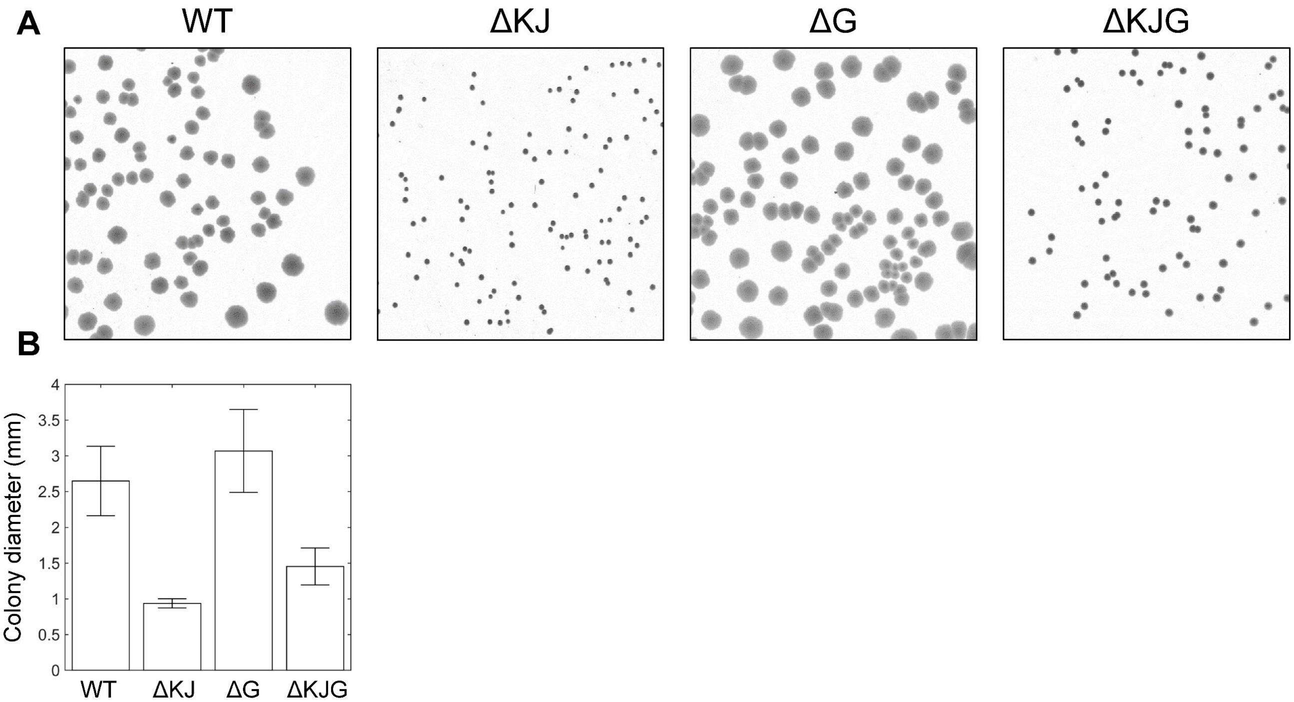

Figure S1 : Quantification of *E. coli* W3110 variant growth at 37°C. Cells were plated at constant density on agar plates without antibiotics and grown overnight at 37°C (panel **A**), and colony diameters were quantified (panel **B**).

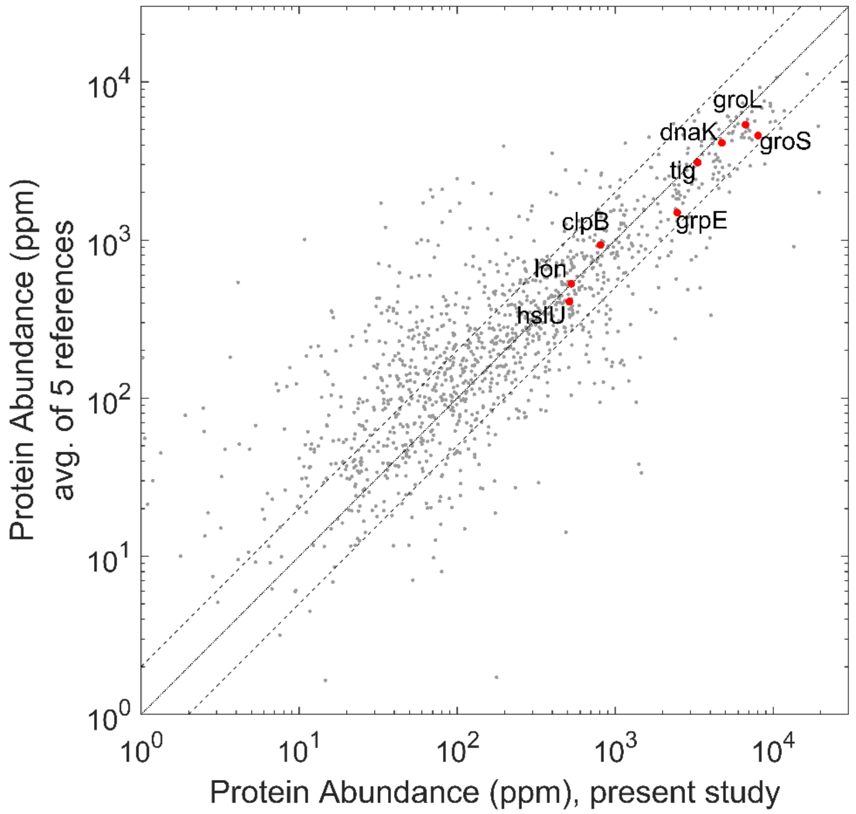

Figure S2: Abundance comparison between the current MS data (y-axis) and the average values from five published *E. coli* proteomic studies (1-5) Published data sets for comparison were obtained from PaxDB or the respective supplementary data files. The scatter plot shows the abundances of 1212 proteins commonly quantified all data sets. The Pearson correlation coefficient is 0.71. The dotted line represents the 1:1 abundance ratio; proteins within the two dashed lines have less than two-fold abundance differences between our data and the other published data sets.

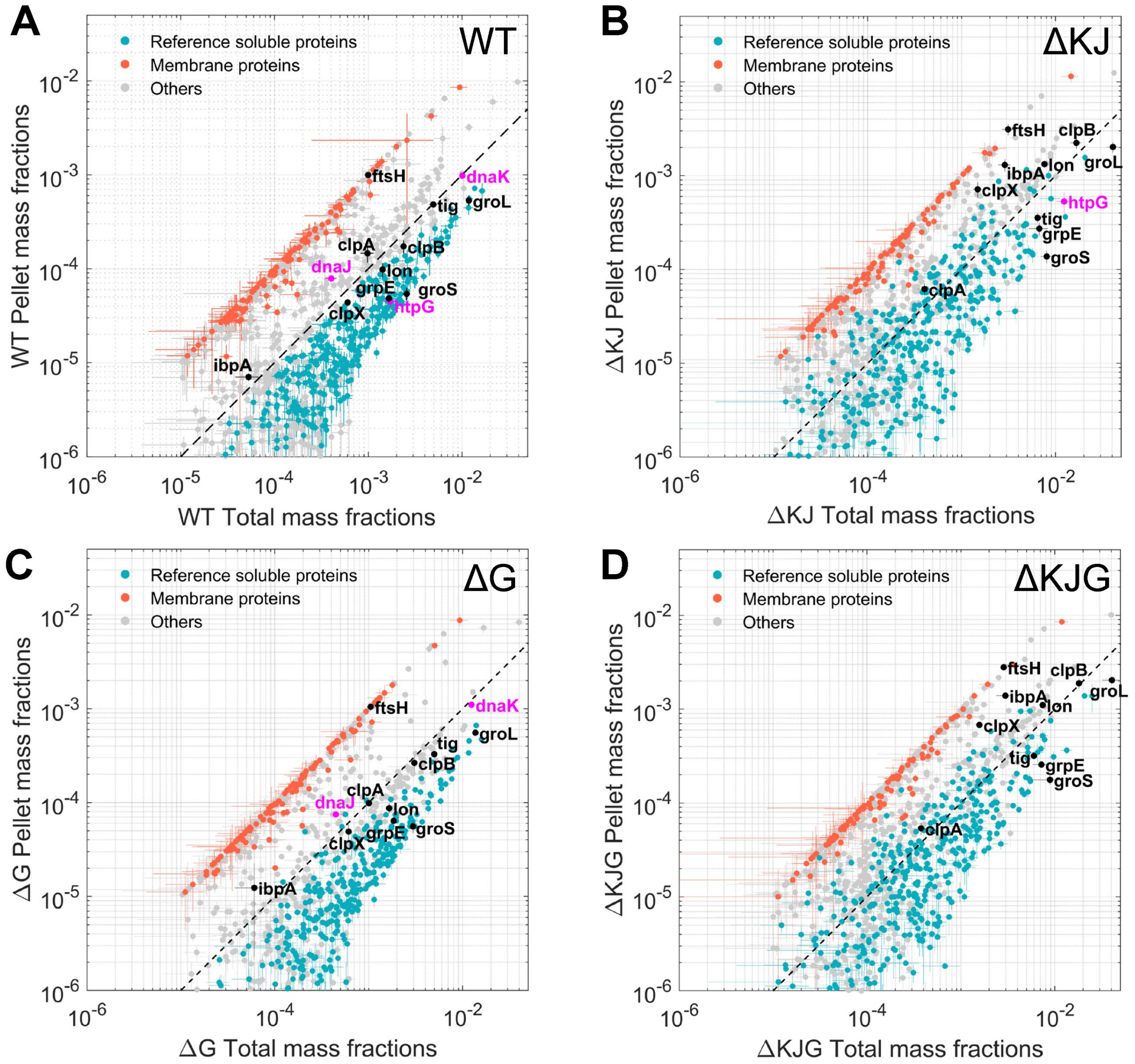

Figure S3 : Normalized total and insoluble (Pellet) mass fraction scatter plots in the four *E. coli* variants : WT (panel **A**), ΔKJ, (panel **B**), ΔG (panel **C**), and ΔKJG (panel **D**). Orange dots: 281 membrane proteins identified as such by the Uniprot database. Blue dots bellow the hatched line: proteins with a significant solubility index equal or greater than 90%. Grey circles: other less significantly soluble proteins. Magenta dots: DnaK, DnaJ and HtpG. Black dots, other classical chaperones and major proteases.

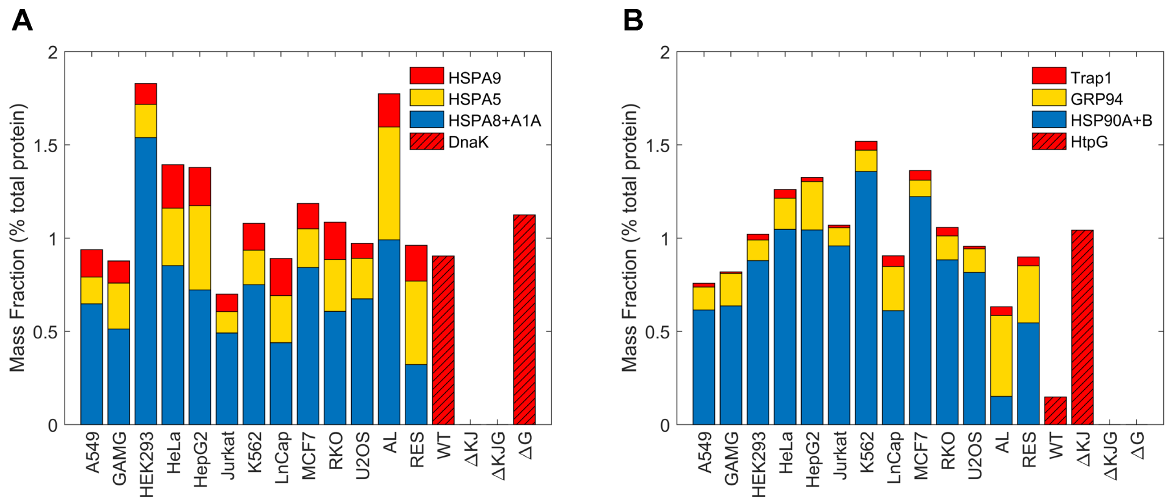

Figure S4: Estimated mass fraction values from quantitative MS analysis, expressed in % of the total cellular proteins, of the main HSP70s (panel **A**) and HSP90s (panel **B**) in immortalized human cell lines (6) and naive rat liver cells stressed by excessive chronic food intake (AL), or non-stressed under mild food-restriction (RES) (7). Chaperone orthologues in different cellular compartments: cytosol (Blue), ER lumen (Yellow), mitochondria (red). Bacterial chaperones are shown with hatched bars.

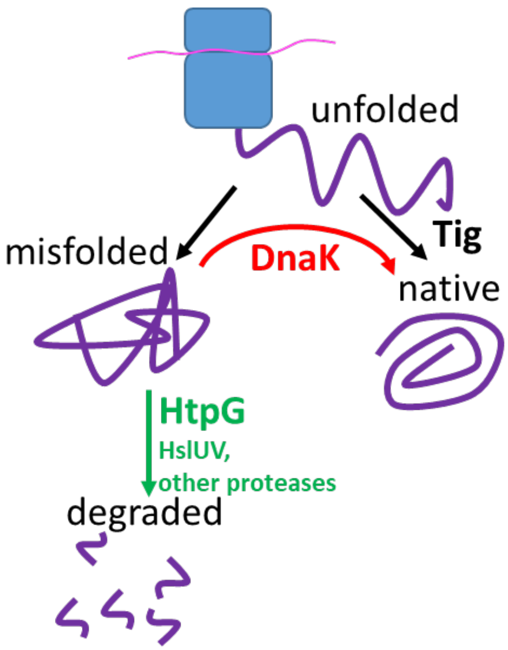

Figure S5: HtpG-directs misfolded DnaKJ-substrates to degradation**.** A subset of unfolded nascent polypeptides exiting the ribosome may be assisted by trigger factor (Tig) at reaching the native state (right black arrow), whereas others that misfold (left black arrow), can be unfolded again by DnaKJ (red arrow) to thereafter fold to the native state (right black arrow). Without DnaKJ, the misfolded species are directed by HtpG to proteases such as HslUV for degradation (green arrow). In the ΔKJ knockout, HtpG and proteases are massively upregulated, leading to an excessive degradation of essential DnaKJ-dependent proteins and limiting bacterial growth at 37°C. Further deletion in the ΔKJ knockout of HtpG, or HslV, reduces excessive degradation of DnaKJ-dependent proteins, allowing some aggregation-prone polypeptides to be converted by the other chaperones into native functional proteins and thus ameliorating growth at 37°C.

Table S1: Intracellular concentrations of chaperones and proteases in the different *E. coli* variants. Average and standard deviations (SD) of micromolar concentrations (five biological replicates) are shown.

	Average concentration (µM)							
	WT		ΔG		ΔKJ		ΔKJΔG	
**Chaperones**	mean	SD	mean	SD	mean	SD	mean	SD
GroS	58.0	5.0	66.9	6.5	181.9	25.5	200.6	72.1
GroL	48.4	2.9	56.0	4.5	167.2	24.3	166.2	34.7
DnaK	34.3	1.8	42.2	2.8	0.0	0.0	0.0	0.0
Tig	24.1	2.2	24.3	1.4	31.4	3.0	29.1	2.1
GrpE	17.9	1.4	19.8	1.1	72.3	16.6	77.5	15.1
ClpB	5.8	0.3	7.5	0.5	40.9	5.7	44.4	2.6
HtpG	5.6	0.9	0.0	0.0	40.5	6.9	0.0	0.0
HslO	4.4	0.6	4.8	0.5	35.4	9.3	31.3	3.2
DnaJ	2.3	0.3	2.6	0.6	0.0	0.0	0.0	0.0
IbpA	0.8	0.2	0.9	0.3	42.9	6.5	44.2	8.8
HscA	0.8	0.2	0.8	0.2	1.6	0.2	1.3	0.3
Spy	0.3	0.0	0.4	0.2	0.2	0.1	0.1	0.2
CbpA	0.2	0.0	0.2	0.1	0.1	0.1	0.1	0.1
DjlA	0.0	0.0	0.0	0.0	0.1	0.0	0.0	0.0
HscB	0.1	0.1	0.3	0.2	0.8	0.1	0.5	0.1
IbpB	0.0	0.0	0.0	0.0	21.0	2.3	21.4	1.6
SurA	2.7	0.1	2.5	0.2	2.8	0.3	2.7	0.4
SecB	38.3	5.0	38.2	4.7	23.0	5.0	27.1	5.9
**Proteases**	mean	SD	mean	SD	mean	SD	mean	SD
OmpT	6.8	1.8	8.7	2.3	11.6	3.2	12.7	2.4
ClpP	8.5	0.7	9.1	0.4	16.8	4.0	16.1	3.3
FtsH	3.3	0.2	3.5	0.3	10.4	1.5	9.5	0.6
DegP	5.1	0.2	4.8	0.4	5.2	0.9	4.4	0.2
HflC	2.7	0.0	2.8	0.1	7.4	1.2	6.8	0.4
HflK	2.2	0.2	2.1	0.4	5.9	1.1	5.1	0.4
Lon	3.8	0.3	4.4	0.3	20.4	1.9	19.9	1.8
HslU	3.7	0.2	4.5	0.3	27.9	3.3	26.9	1.1
ClpX	3.1	0.1	3.1	0.2	7.5	0.8	8.0	0.4
LoiP	1.3	0.1	1.0	0.1	1.4	0.2	1.2	0.3
ClpA	2.7	0.4	2.8	0.1	1.1	0.2	1.1	0.1
HslV	2.1	0.5	2.6	0.5	18.8	2.5	13.9	1.9
HtpX	1.0	0.1	1.0	0.1	3.3	0.6	3.3	0.2
PmbA	1.6	0.1	1.6	0.1	1.9	0.1	1.6	0.2
YcbZ	0.5	0.0	0.5	0.0	0.8	0.1	0.7	0.0
TldD	0.7	0.2	0.7	0.2	0.8	0.1	0.7	0.1
DegQ	0.5	0.0	0.5	0.1	0.3	0.0	0.3	0.1
DegS	0.2	0.0	0.2	0.0	0.3	0.0	0.2	0.1

Additional dataset: table S2 (separate file)

Table S2: Absolute quantities of 1339 significantly quantified proteins in the four E. coli variants, expressed as mass fractions (sheet 1) and molarity (µM, sheet 2). Numbers show means and standard deviations for five biological replicates.

Additional dataset: table S3 (separate file)

Table S3: Normalized pellet mass fractions (means & standard deviations, five biological replicates), indicating the quantities of insoluble proteins for each of the 1339 significantly quantified proteins in the four *E. coli* strains.

Additional dataset: table S4 (separate file)

Table S4: Proteins with statistically significant (FDR < 0.05) solubility difference between WT and KJ, and with a solubility difference larger than 10 percent points.

References

1. Craig R, Cortens JP, & Beavis RC (2004) Open source system for analyzing, validating, and storing protein identification data. *J Proteome Res* 3(6):1234-1242.
2. Valgepea K*, et al.* (2010) Systems biology approach reveals that overflow metabolism of acetate in Escherichia coli is triggered by carbon catabolite repression of acetyl-CoA synthetase. *BMC Syst Biol* 4:166.
3. Arike L*, et al.* (2012) Comparison and applications of label-free absolute proteome quantification methods on Escherichia coli. *J Proteomics* 75(17):5437-5448.
4. Krug K*, et al.* (2013) Deep coverage of the Escherichia coli proteome enables the assessment of false discovery rates in simple proteogenomic experiments. *Mol Cell Proteomics* 12(11):3420-3430.
5. Schmidt A*, et al.* (2016) The quantitative and condition-dependent Escherichia coli proteome. *Nat Biotechnol* 34(1):104-110.
6. Finka A & Goloubinoff P (2013) Proteomic data from human cell cultures refine mechanisms of chaperone-mediated protein homeostasis. *Cell stress & chaperones* 18(5):591-605.
7. Gat-Yablonski G*, et al.* (2016) Quantitative proteomics of rat livers shows that unrestricted feeding is stressful for proteostasis with implications on life span. *Aging* 8(8):1735-1758.
